# Automated 3D sheep posture estimation and telemetric pressure synchronization via a markerless computer vision pipeline

**DOI:** 10.3389/fvets.2026.1933870

**Published:** 2026-10-14

**Authors:** Martina Roncoroni, Anthony Podgoršak, Andreas Kaufmann, Fabian Flürenbrock, Nina Eva Trimmel, Markus Florian Oertel, Melanie Zeilinger, Miriam Weisskopf, Marianne Schmid Daners

**Affiliations:** 1 Department of Mechanical and Process Engineering, Institute for Dynamic Systems and Control, ETH Zurich, Zurich, Switzerland; 2 Product Development Group Zurich, Department of Mechanical and Process Engineering, ETH Zurich, Zurich, Switzerland; 3 Center for Preclinical Development, University Hospital Zurich, University of Zurich, Zurich, Switzerland; 4 Department of Neurosurgery, University Hospital Zurich, University of Zurich, Zurich, Switzerland

**Keywords:** animal models, cerebrospinal fluid dynamics, computer vision, pose, telemetric pressure recordings, training

## Abstract

Body posture is a key determinant of multi-compartmental pressure dynamics, yet continuous posture monitoring in chronic large animal studies remains technically challenging. To investigate physiological pressure dynamics as a function of body posture in an ovine model, we present a markerless computer vision pipeline that calculates three-dimensional (3D) pose estimates of an individual sheep during training sessions and automatically synchronizes posture with telemetric pressure recordings. First, two-dimensional (2D) joint localization was performed using a combination of Faster R-CNN and Lite-HRNet, achieving a mean average precision (mAP) of 77.4%. Second, we triangulated the detected 2D keypoints into 3D space and leveraged a skeletal and temporal prior to mitigate 2D localization errors. Third, we computed anatomical angles and used a multilayer perceptron to classify five structured training postures (standing, walking up a bridge, walking down a bridge, kneeling, sitting) achieving an overall accuracy of 95.2% on a held-out test set of 647 frames drawn from six sheep, and a leave-one-subject-out cross-validated accuracy of 68.8% when tested on animals unseen during training. Finally, the telemetric recordings of physiological pressures are synchronized to the respective posture allowing for retrospective analysis of body posture in relation to pressure dynamics. This pipeline lays the foundation for future investigations into posture-dependent pressure recordings, with implications for both animal modeling and clinical translation to humans.

## 1 Introduction

Body posture markedly influences intracranial, cardiovascular, and abdominal pressures. Understanding how posture influences these pressures and their interactions between compartments is critical to interpreting pressure measurements and developing realistic models of animal or human physiology. However, such investigations in humans remain limited in scope because ethical considerations constrain the feasibility of long-term invasive measurements across multiple pressure compartments. Animal models therefore provide a viable means to investigate posture-dependent pressure dynamics across compartments. Specifically, sheep studies have been used to investigate physiological conditions and to derive baseline values of cerebrospinal fluid dynamics (1–3). Sheep are comparable to humans in body weight, and the intracranial and vascular pressure ranges of sheep are comparable to those observed in humans (1). When properly handled, sheep can be placed into a specially designed chair that allows them to sit upright (2). Furthermore, their jugular veins exhibit posture-dependent diameter changes, indicating behavior similar to that observed in humans, making venous dynamics comparable despite their quadrupedal posture (3). All of these characteristics support the comparison with human dynamics.

Prior work in human literature has established the physiological relationships that motivate this investigation. The concept of the hydrostatic point of indifference was originally studied in relation to cardiovascular regulation in humans (4) and was later extended to include effects on pulmonary function (5), cerebral venous blood flow (6), and intracranial pressure (ICP) dynamics in human models (7). More recently, long-term telemetric measurements in human subjects have demonstrated that body posture is a major determinant of ICP and a critical confounder in the interpretation of pressure recordings even under everyday conditions (8). These findings collectively motivate the investigation of analogous posture-dependent pressure relationships in the ovine model, where multi-compartment measurements can be performed longitudinally and invasively. Given the coupling between body posture and physiological pressure, continuous monitoring of the animal's posture is essential for correctly interpreting pressure recordings collected during extended experiments.

Computer vision provides a means to quantify animal pose continuously and non-invasively. Methods that leverage motion capture systems, which employ physical markers or inertial sensors on the animal to track body kinematics, have previously been used to perform this task (9, 10). However, these methods require the attachment of devices to the fur, which may cause discomfort and interfere with the natural behavior of the animal. Therefore, such approaches are generally less suitable for long-term monitoring, especially in studies that require natural, undisturbed movements and a low level of stress for the animal.

Recent advances in deep learning have led to the emergence of markerless approaches that derive body posture directly from video data, enabling objective quantification of animal movement and pose without interfering with natural behavior (11–13). These methods rely on trained convolutional neural networks to detect body keypoints (KPs) using annotated image datasets. Social Long-term Effective Any Point (SLEAP) tracking demonstrated high-precision two-dimensional (2D) tracking of body parts in freely moving rodents, achieving subpixel accuracy and allowing fine-grained behavioral quantification (11). DeepLabCut introduced a generalizable framework for markerless tracking of user-defined body parts across a range of species (12). SuperAnimal, a pre-trained cross-species model included in DeepLabCut, enables the transfer of learned representations between different animals and datasets (14). These advances have markedly reduced the annotation burden and increased generalizability in animal pose estimation.

However, estimating animal posture in realistic environments remains challenging due to self-occlusions, variable lighting, and complex body configurations that degrade the accuracy of KP detection. To overcome these limitations, recent methods have begun integrating temporal information to improve stability across frames. For instance, OptiFlex (15) combines deep learning-based KP detection with optical flow to enforce temporal coherence and refine animal pose estimation over a sequence of frames in a single-camera setup. Similarly, the method presented in Acinoset3D (16) has addressed these issues by introducing multi-camera recordings and temporal models that take advantage of the continuity between frames to improve the robustness under occlusion.

A growing body of work has applied markerless pose estimation specifically to livestock. Sheep-focused studies have addressed behavior and posture classification from 2D video (17), and lightweight real-time posture recognition (18). These approaches, however, operate in 2D. Separately, 3D markerless pose estimation has advanced substantially in livestock applications: MAMMAL reconstructs 3D surface motion of multiple freely moving pigs via articulated mesh alignment, remaining robust under the severe occlusions typical of social large-animal housing (19). To date, no pipeline exists that simultaneously estimates 3D body posture markerlessly and synchronizes it with multi-compartment telemetric pressure recordings in a chronic ovine model. Building on the recent developments in the field of markerless KP estimation and trajectory-smoothing techniques, we propose a pipeline to capture the three-dimensional (3D) posture of sheep using stereo video data during training, to synchronize these five structured movements that comprise standing, walking up and down a bridge, kneeling and sitting with telemetric pressure recordings to enable the investigation of posture-dependent pressure dynamics in sheep. This framework is intended to make longitudinal pressure data from chronic ovine trials interpretable in the context of body posture, with implications for both large animal physiology and the clinical translation of findings to human pressure regulation and cerebrospinal fluid dynamics research.

## 2 Method

The methods presented in this study describe the complete data acquisition and processing pipeline developed to estimate the posture of the sheep and synchronize these measurements with continuously recorded physiological pressure signals. This modular pipeline integrates synchronized stereo video acquisition, telemetric pressure recordings, and a computer vision workflow consisting of 2D KP detection, 3D reconstruction, trajectory smoothing, anatomical angle computation, and posture classification. A schematic overview of the pipeline is shown in Figure 1.

**Figure 1:**
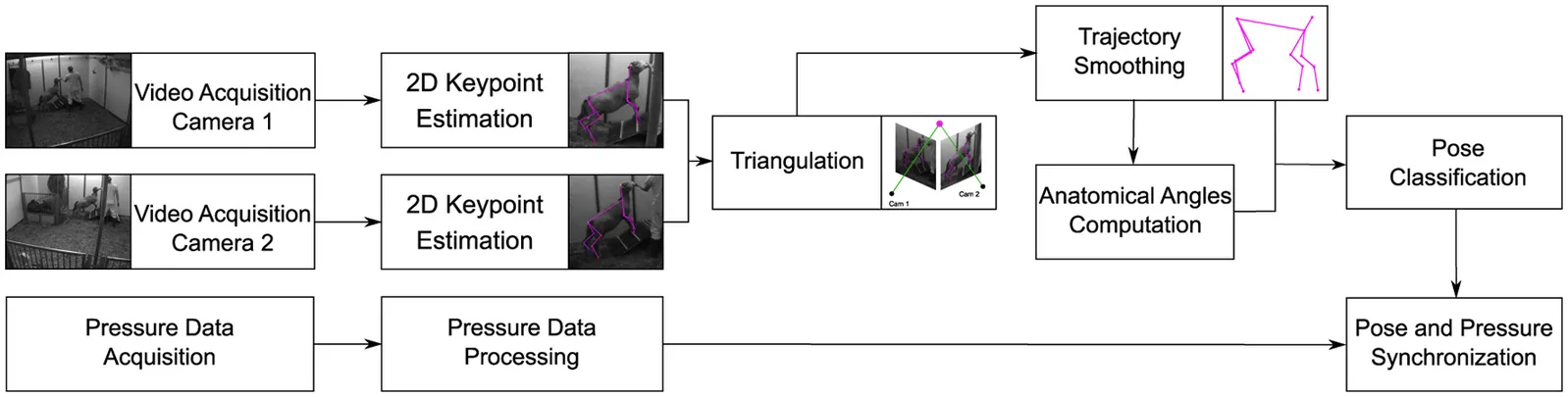
Visual overview of the computer vision analysis pipeline. Video and pressure data were acquired, and the video data were then fed into the pipeline for keypoint estimation. The 2D images were then triangulated into 3D, smoothed, and the relevant body angles of interest calculated. A classifier was used to derive the pose and finally all images were synchronized to the telemetric pressure data.

### 2.1 Animals and ethical statement

Six female white Alpine sheep (Ovis gmelini aries) were considered in this study. All sheep were mature (2–5 years old) and weighed an average of 79.5 ± 8.0 kg. Animals were housed in groups of 2-4 animals and given a minimum of 2 weeks acclimation period. All health status and housing complied via formal attestation with the Swiss Federal Food Safety and Veterinary Office (FSVO) regulations. Animal housing and all experimental procedures were approved by the local Committee for Experimental Animal Research (Cantonal Veterinary Office Zurich, Switzerland) under the license number ZH119/2019, and were conforming to the European Directive 2010/63/EU of the European Parliament and the Council on the Protection of Animals used for Scientific Purposes, as well as to the Guide for the Care and Use of Laboratory Animals (20).

### 2.2 Anesthesia and animal preparation

Sheep underwent surgical implantation of radio-telemetric pressure sensors (Stellar implantable transmitters, TSE Systems GmbH). Prior to surgery, sheep were fasted for 16–18 h, with unrestricted access to water during the fasting period. The animal was pre-medicated with 3 mg/kg body weight (BW) ketamine hydrochloride (Ketasol^®^ 100, Dr. E. Graeub AG) and 0.3 mg/kg BW midazolam (Dormicum^®^ Roche Pharma) intravenously. As prophylaxis, each animal received a subcutaneous injection of 3 mL tetanus serum (Tetanus-Serum Intervet, ad us. vet., MSD Animal Health GmbH). Peri-operative antibiotic treatment consisted of intravenous sodium penicillin at 30,000 IU/kg BW (Penicillin Natrium Streuli^®^ ad us. vet., Streuli Tiergesundheit AG) and gentamycin (4 mg/kg BW) (Vetagent^®^ ad us. vet., MSD Animal Health GmbH). Before orotracheal intubation, 2–5 mg/kg BW of propofol (Propofol-^®^ Lipuro 1%, B. Braun Medical AG) was administered intravenously. Anesthesia was maintained using inhaled isoflurane (Attane™ Isoflurane ad us. vet., Piramal Enterpr. India) in an oxygen/air mixture, balanced with a constant-rate infusion of propofol (Propofol-^®^ Lipuro 1%, B. Braun Medical AG). Intraoperative analgesia was provided via a continuous intravenous infusion of sufentanil (Sufenta^®^ forte, Janssen-Cilag AG; 2.5 μg/kg BW/h i.v.). Before each surgical incision, deposits of 5–10 mL of the local anesthetic ropivacaine (Ropivacain Sintetica 1%, Sintetica S.A.) was administered subcutaneously at the respective sites. Post-operatively, antibiotic treatment and multimodal analgesia were continued for 5 days.

### 2.3 Animal housing and training protocol

The primary application of the developed pipeline is to estimate the posture of the sheep and synchronize the calculated 3D anatomical KPs with telemetrically recorded pressure signals (Figure 2). During the chronic trials, the sheep move freely in a 15 m^2^ stable (Figure 3). Structured training was conducted over the course of at least one month for each of the six sheep. For animal welfare, at least one companion sheep was housed with the experimental subject throughout the trial period. To capture data across a wide range of postures, a trainer guided the sheep through specific movements, including standing, walking up and down a bridge, kneeling, and sitting in a chair (Figure 4). During these guided pose training sessions, the companion sheep was temporarily relocated to an adjacent side stable to avoid interference.

**Figure 3:**
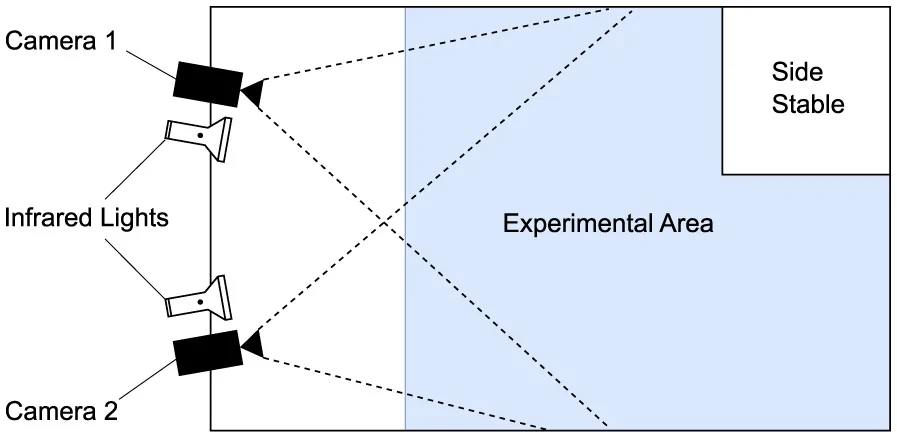
Schematic of the experimental setup. Two cameras and their respective infrared lamps are positioned to provide a stereoscopic view of the experimental area. The field of view of each camera is marked with dashed lines. The side stable serves as a designated area to temporarily house the accompanying sheep during the position exercises.

**Figure 2:**
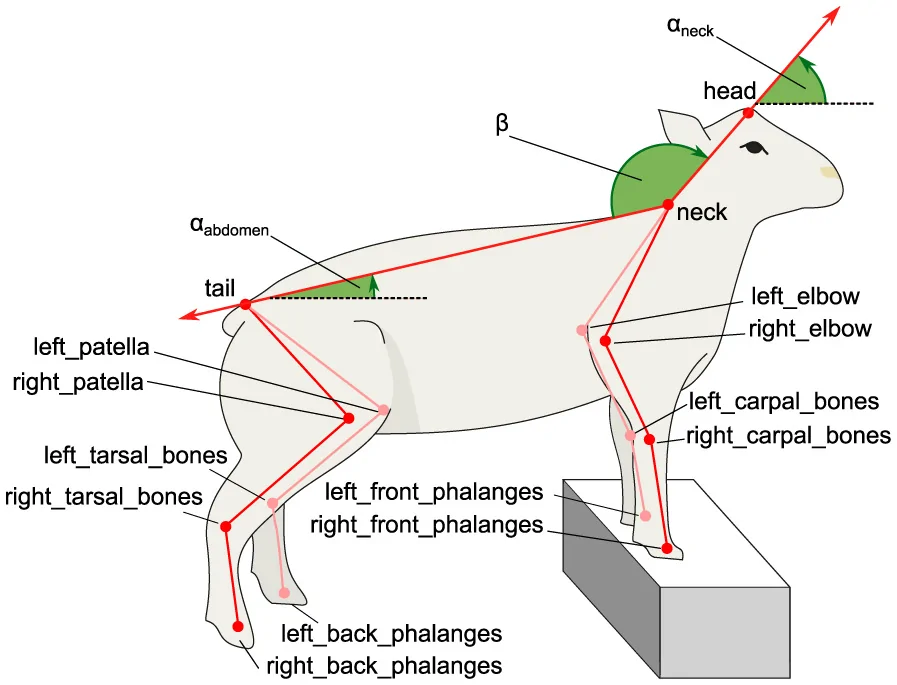
Definition of keypoint locations and body angle representations used for sheep posture analysis. Angle α_abdomen_ represents the absolute angle between the abdomen and the ground. Angle α_neck_ denotes the absolute angle between the neck and the ground. Angle β describes the relative angle between the neck and the abdomen.

**Figure 4:**
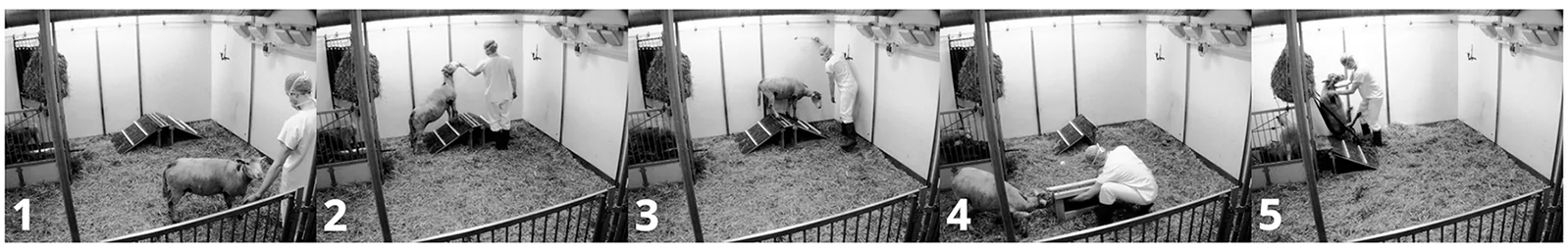
Exemplary images of the five pose categories for classification. From left to right: **(1)** standing, **(2)** walking up, **(3)** walking down, **(4)** kneeling, and **(5)** sitting in chair.

### 2.4 Video data acquisition

Videos were acquired using a monochrome stereoscopic power-over-Ethernet (PoE) surveillance system (Blackfly S GigE, Teledyne FLIR LLC) with two cameras, set to operate at three frames per second (FPS). Precise synchronization between the two cameras was ensured by fixed cabling via a general-purpose input/output cable (ACC-01-3010, FLIR Integrated Imaging Solutions Inc.) with camera 1 set as master and camera 2 as slave. The cameras were connected to a gigabit network Ethernet host adapter card (GIGE-PCIE2-2P02, FLIR Integrated Imaging Solutions Inc.) directly on the motherboard of a central workstation via Cat7 RJ45 cables. Image pairs were acquired and visualized via Spinnaker SDK (Teledyne FLIR LLC) and saved locally on the workstation. Finally, two automatically activating 850 nm LED array and 45° beam angle infrared panel lamps (NightVisionExperts) were used to support night time observation.

### 2.5 Pressure data acquisition

Arterial blood pressure (ABP) was measured via a sensor implanted in the carotid artery, while central venous pressure (CVP) was monitored through a sensor placed in the right common jugular vein. Intracranial pressure (ICP) was recorded using a sensor positioned in the right lateral cerebral ventricle. Intrathecal pressure (ITP) was measured via an intrathecal sensor inserted between the L6–L7 spinal vertebrae. The remaining four sensors were distributed throughout the abdominal cavity to monitor intra-abdominal pressure (IAP) at specific sites: cranial (IAP_Cranial_), caudal (IAP_Caudal_), dorsal (IAP_Dorsal_), and ventral (IAP_Ventral_).

All pressure recordings were acquired telemetrically at 100 Hz using Notocord (Notocord Hem, Instem). Each implant consists of solid-state pressure sensor-tipped catheters with an effective measurement range of −25 to 300 mmHg, a resolution of 0.10 mmHg with an additional temperature recording resolution of 0.05 °C. In addition, infrared light sensors were included on every implant to allow for online data synchronization accounting for the separate internal clocks of each implant. Further implantation details can be found in Podgoršak et al. (2).

For ICP and ITP, single-channel glass-tipped implants (Type PTA-I2 D-430001-IMP-15) were implanted with measurement catheters extending into the intracranial and lumbar cerebrospinal fluid spaces to ensure direct fluid pressure measurements. For the measurement of ABP and CVP, a dual-channel implant with on-board electrocardiogram (ECG) leads (Type PPBTA-XL titanium-tipped) were used. For abdominal measurements, a quad-channel implant (Type PPPPTA-XXL titanium tip) was initially used (sheep No. 1 and 2) but was later replaced with two smaller dual-channel implants (Type PPBTA XL titanium tip) to reduce scar tissue formation around implants (sheep No. 3–6). Both types of sensors provided measurements of IAP at the four locations in the abdomen.

### 2.6 Pressure data processing

Pressure data are processed as follows. First, signal dropouts are removed: a lower limit for the permissible validity of the data is defined in accordance with the sensor manufacturer's recommendation by subtracting 50 mmHg from the average of the entire data series, and all signal recordings below this threshold are removed. Outliers are then removed using a one-second moving window: all pressure measurements that deviate from the median by more than three standard deviations within this window are identified as an outlier and excluded.

### 2.7 2D keypoint estimation

In order to track the 3D position of the sheep using computer vision, 15 KPs, which represent anatomical locations on the sheep, are selected to ensure a comprehensive representation of the animal as an ideal rigid skeleton (Figure 2). A top-down KP estimation approach is used, consisting of two sequential steps: the sheep is first detected in each image using a bounding-box detector; subsequently, KPs are estimated within the detected region. For sheep detection, we employ Faster R-CNN (21), the output of which is a bounding box containing the sheep, afterwards 2D KP estimation is performed within the detected bounding box using the Lite-HRNet (22) single subject pose estimation model. This heatmap-based KP detection architecture is used for estimating the pose within the detected regions of interest, which are the bounding boxes. The dataset is manually annotated to train the selected computer vision model, using the Common Objects in Context (COCO)-Annotator (23). Animal images are annotated by a bounding box, specified as [*x*,*y*,*w*,*h*] (the pixel coordinates of the top left corner (*x*,*y*), together with the width w and height h) and 15 body KPs, each represented by an image coordinate (*x*,*y*) and a visibility flag *v*∈{0, 1, 2} indicating whether the KP is 0 not labeled, 1 labeled but not visible, or 2 labeled and visible. The manually labeled data set for the 2D KP estimate consist of 1,916 images, split into 1,867 training and 47 held-out test images. To mitigate overfitting, model performance is monitored using validation loss. At the beginning of each training run, the dataset is randomly split into training and validation subsets, with 10% of the training data reserved for validation.

The performance of the 2D KP and bounding box detections is evaluated using Average Precision (AP), as in the standard COCO bounding-box evaluation protocol (24). Higher values of AP indicate better performance, with 100% representing perfect detection. For performance evaluation of 2D KP detection, AP is computed based on Object KP Similarity (OKS) (24) across multiple OKS thresholds ranging from 0.50 to 0.95 in steps of 0.05 [denoted AP @ (0.5:0.95)], as well as at thresholds 0.5 (AP@0.5) and 0.75 (AP@0.75). OKS is a metric that measures how well predicted KPs align with ground-truth KPs and is defined as in Equation 1:

$$\begin{array}{l} OKS\;=\frac{\sum_{i}\left[\;exp\left(\;\frac{-d_{i}^{2}}{2s_{i}^{2}\kappa_{i}^{2}}\right)\delta \left(v_{i}\;>\;0\right)\;\right]}{\sum_{i}\left[\delta \left(v_{i}>0\right)\right]}, \end{array}$$ (1)

where *d*_*i*_ is the Euclidean distance between the predicted point and the ground truth, *s*_*i*_ is the scale value as the square root of the boundary box area and κ_*i*_ is a constant that controls the tolerance of localization for each KP.

### 2.8 Triangulation

To reconstruct the joint locations of the KPs in 3D, a multiview triangulation is performed. This is essential for the correct calculation of the anatomical angles and the estimation of body posture. Intrinsic parameters, including camera matrices and distortion coefficients, are obtained using the OpenCV (25) calibration toolkit with a custom checkerboard pattern. For extrinsic calibration, which determines the relative locations of the cameras in the 3D space, we employ MASt3R (26), a deep learning model for stereo matching, using the pre-trained model and the default hyperparameters. The 3D locations of the joints are then calculated by applying the OpenCV function triangulatePoints for each pair of frames in the video sequence.

### 2.9 Trajectory smoothing

The triangulated 3D joint positions are not recovered when a joint is visible in less than two camera views; moreover, minor discrepancies in the KP localization across views, where the predicted pixel coordinates do not precisely correspond to the same anatomical landmark, can lead to artifacts in the 3D triangulation. To overcome these inaccuracies, Acinoset3D provides a method to enforce both temporal and structural consistency in 3D pose reconstruction (16). In our implementation, the original Acinoset3D approach is modified by reducing the number of joints in the skeleton model to 15, as the number of KPs estimated in Section 2.7. A kinematic model is built by providing a prior on the lengths of each limb of the sheep. In this framework, the state of the system at each time step comprises the joint coordinates, their velocities, and accelerations. The joint coordinates are initially estimated using the 3D triangulated data from Section 2.8, serving as a starting point for further refinement.

Let ${\text{x}}_{j,t}\in {\mathbb{R}}^{d}$ denote the pose-parameter vector at frame *t* for joint *j*. The 3D coordinates of the 15 anatomical keypoints are obtained via a fixed kinematic chain (Equation 2):

$$\begin{array}{l} p_{t}=\Phi \left(x_{j,t};\ell \right), \end{array}$$ (2)

where Φ(·) recursively computes each keypoint's position by rotating a fixed-length segment from its parent joint, and ***ℓ*** are the calibrated, per-animal limb lengths. The trajectory is estimated by solving:

$$\begin{array}{l} \underset{x,\overset{{}^{\circ}}{x},\ddot{x},w}{\min}\;\overset{T}{\underset{t=1}{\sum }}\overset{2}{\underset{i=1}{\sum }}\overset{15}{\underset{j=1}{\sum }}C\left(\|\Pi_{i}\;K_{i}\left(\Phi_{j}\left(x_{j,t};\ell \|\right)\right)\;-u_{i,j,t}\right)+\\ \lambda \overset{T}{\underset{t=2}{\sum }}\overset{15}{\underset{j=1}{\sum }}\|{w_{j,t}}^{2}\|, \end{array}$$ (3)

$$\begin{array}{l} \text{s.t.}\;x_{j,t}=x_{j,t-1}+\Delta t{\overset{•}{x}}_{j,t},t=2,\ldots ,T, \end{array}$$ (4)

$$\begin{array}{l} {\overset{•}{x}}_{j,t}={\overset{•}{x}}_{j,t-1}+\Delta t{\ddot{x}}_{j,t},t=2,\ldots ,T, \end{array}$$ (5)

$$\begin{array}{l} {\ddot{x}}_{j,t}={\ddot{x}}_{j,t-1}+w_{j,t},t=2,\ldots ,T, \end{array}$$ (6)

where *i* indexes the two camera views, Φ_*j*_(·) denotes the *j*-th keypoint's coordinates returned by Φ(·), Π_*i*_(·) projects a 3D point into camera *i*'s image plane, *K*_*i*_(·) transforms world coordinates into camera *i*'s frame, and **u**_*i, j, t*_ is the observed 2D location of joint *j* in camera *i* at frame *t*. Equations 3–6 implement implicit Euler integration linking pose parameters, their velocities, and accelerations across consecutive frames, following Joska et al. (16). *C*(·) denotes the redescending cost function as described in Joska et al. (16), and $\lambda =\frac{1}{\sigma_{model_{j}}}$, where σ_*mode*_*l*__*j*__ is the acceleration variance in camera *j*, normalizes the acceleration disturbance **w**_*j, t*_.

### 2.10 Anatomical angles computation

Postural changes primarily influence pressure dynamics in the body through hydrostatic pressure shifts. Quantifying these effects requires features that capture the orientation of the body relative to gravity. In our pipeline, anatomical joint angles serve as interpretable descriptors of body configuration that reflect physiologically meaningful posture changes (Figure 2).

With this pipeline, we extract three anatomical angles that characterize the orientation of the upper body: the abdomen absolute angle (α_*abdomen*_), neck absolute angle (α_*neck*_), and neck-abdomen relative angle (β). α_*abdomen*_ is defined as the angle between the ground and the vector connecting the tail to the neck. It ranges from −90° to +90°, where positive values indicate that the neck is positioned higher than the tail. α_*neck*_ is calculated similarly, as the angle between the ground and the vector from neck to head. It follows the same range and sign convention, with positive values indicating that the head is higher than the neck. β measures the angle between the tail-to-neck and neck-to-head vectors. This angle is independent of the orientation of the ground and reflects the relative flexion or extension of the neck with respect to the torso along the gravitational axis. In order to compute the anatomical angles, the floor orientation had to be extracted; this was done from the MASt3R-derived scene point cloud using RANSAC (27). At each iteration, three points were sampled at random and a candidate plane was fit through them; the plane was scored by the fraction of remaining scene points lying above it, and the highest-scoring plane's inlier set was retained. The floor normal **f**_⊥_ was then obtained by singular value decomposition on this inlier set, with the in-plane axis **f**_∥_ taken as the direction of dominant variance within the plane.

The angles α_*abdomen*_ and α_*neck*_ were calculated as described in Equation 7:

$$\begin{array}{l} \alpha_{x}=90^{•}-\arccos\left(\frac{u_{x}\cdot f_{⊥}}{∥u_{x}∥\cdot ∥f_{⊥}∥}\right), \end{array}$$ (7)

where **u**_*x*_ represents the vector from the neck to the head (*x*=neck) or from the tail to the neck (*x*=abdomen). The relative angle between the neck and abdomen is calculated by projecting each **u**_*x*_ onto the 2D plane defined by **f**_⊥_ and **f**_∥_ (Equations 8, 9):

$$\begin{array}{l} u_{abdomen}^{2D}=\left(\begin{array}{c} u_{abdomen}\cdot f_{∥}\\ u_{abdomen}\cdot f_{⊥} \end{array}\right), \end{array}$$ (8)

$$\begin{array}{l} u_{neck}^{2D}=\left(\begin{array}{c} u_{neck}\cdot f_{∥}\\ u_{neck}\cdot f_{⊥} \end{array}\right). \end{array}$$ (9)

The relative neck-abdomen angle β was computed using a single plane defined by the neck (**p**_0_), tail (**p**_*u*_), and head (**p**_*v*_) keypoints. Direction vectors were defined as **u** = **p**_*u*_−**p**_0_ and **v** = **p**_*v*_−**p**_0_. An orthonormal in-plane basis was constructed via Equation 10:

$$\begin{array}{l} {\hat{e}}_{u}=\frac{u}{∥u∥},\;{\hat{e}}_{v}=\frac{v-\left({\hat{e}}_{u}\cdot v\right){\hat{e}}_{u}}{∥v-\left({\hat{e}}_{u}\cdot v\right){\hat{e}}_{u}∥}, \end{array}$$ (10)

By construction, the tail lies on the positive ${\hat{\text{e}}}_{u}$ axis (θ_*u*_ = 0), and the head's in-plane angle is computed as shown in Equation 11:

$$\begin{array}{l} \theta_{v}=\text{atan}2\left(v\cdot {\hat{e}}_{v},\;v\cdot {\hat{e}}_{u}\right). \end{array}$$ (11)

Because θ_*v*_ alone does not disambiguate which of the two angular regions bounded by ${\hat{\text{e}}}_{u}$ and **v** corresponds to the physical torso flexion, a fourth reference keypoint on the lower body, **p**_*s*_, was projected onto the same basis to obtain $\theta_{s}=\text{atan}2\left(\left({\text{p}}_{s}-{\text{p}}_{0}\right)\cdot {\hat{\text{e}}}_{v},\;\left({\text{p}}_{s}-{\text{p}}_{0}\right)\cdot {\hat{\text{e}}}_{u}\right)$. The side-disambiguating reference point **p**_*s*_ was taken as the centroid of a set of lower-body keypoints (Equation 12):

$$\begin{array}{l} p_{s}=\frac{1}{\left|S\right|}\underset{k\in S}{\sum }p_{k}, \end{array}$$ (12)

where S comprises the twelve limb keypoints. The relative angle β was then taken as the angle on the side of ${\hat{\text{e}}}_{u}$ opposite **p**_*s*_ (Equation 13):

$$\begin{array}{l} \beta =\{\begin{array}{cc} 2\pi -\theta_{v} & \text{if}\;0\leq \theta_{s}\leq \theta_{v}\\ \theta_{v} & \text{if}\;\theta_{s}<0\leq \theta_{v}\;\text{or}\;0\leq \theta_{v}<\theta_{s}\\ 2\pi +\theta_{v} & \text{if}\;\theta_{v}\leq \theta_{s}\leq 0\\ -\theta_{v} & \text{if}\;\theta_{v}\leq 0<\theta_{s}\;\text{or}\;\theta_{s}<\theta_{v}\leq 0. \end{array} \end{array}$$ (13)

### 2.11 Pose classification

The posture of the sheep is classified into one of five predefined categories, with examples shown in Figure 4: standing, walking up, walking down, kneeling, and sitting in a chair. A multilayer perceptron (MLP) is used for this purpose, taking as input the 3D coordinates of the joints expressed relative to the head location, with the rotation about the gravitational axis normalized, together with the three anatomical angles α_*neck*_, α_*abdomen*_, β, yielding 45 input features. The head-relative representation sets the head coordinates to zero, reducing the number of input features by three. The MLP topology and regularization hyperparameters were selected through a randomized search over 30 candidate configurations, evaluated by five-fold stratified cross-validation on the training set using macro-averaged F1 score as the optimization criterion. The search space covered hidden-layer widths in {(64), (128), (128, 64), (256, 128), (128, 64, 32)}, L2 regularization strengths α∈{10^−4^, 10^−3^, 10^−2^}, and initial learning rates in {10^−4^, 5 × 10^−4^, 10^−3^}. The labeled dataset comprised 3,234 frames drawn from 18 recording sessions across all animals. A 80/20 split was applied to obtain a training set of 2,587 frames and a held-out test set of 647 frames, with class proportions preserved in both partitions.

### 2.12 Pose and pressure synchronization

After estimating anatomical angles and body positions, these are synchronized with the pressure recordings. Both camera frames and pressure sensor data are acquired with corresponding timestamps, enabling direct synchronization. Given the different sampling frequencies of the video (3 FPS) and pressure recordings (100 Hz), the 3D KP estimation and angles are linearly interpolated and upsampled to 100 Hz. Linear interpolation assumes that joint motion is uniform between consecutive frames; the maximum temporal uncertainty on the joint localization introduced by this assumption is ±Δ*t*/2 = ±167 ms, occurring at the midpoint between two frames.

### 2.13 Computational environment

This pipeline is implemented on a Linux Ubuntu 22.04.6 operating system with an AMD Ryzen 9 5900X 12-core processor running at 4.12 GHz, 64 GB RAM and a NVIDIA GeForce RTX 3080 GPU (NVIDIA Corporation). The 2D keypoint detection is implemented in Python 3.7 with PyTorch 1.10.1, torchvision 0.11.2, CUDA 11.1 (NVIDIA Corporation), mmdetection 2.25.0, mmpose 0.26.0. The calibration, full trajectory estimation, angle calculation, pose classification and pressure synchronization modules are implemented in Python 3.12.3 (28), PyTorch 2.13.0 (29), torchvision 0.28.0 (29), CUDA 13.0.3. The selected MASt3R checkpoint was “naver/MASt3R_ViTLarge_BaseDecoder_512_catmlpdpt_metric”, with 512 px input, 300 iterations, cosine LR schedule, lr = 0.01. The trajectory smoothing NLP is formulated with Pyomo 6.10.1 (30) and solved with IPOPT 3.14.19 (31).

## 3 Results

An exemplary output of the proposed pipeline is presented in Figure 5. It illustrates how multimodal data streams are aligned along a common temporal axis, allowing for a direct comparison between 3D joint locations, body angles, and posture with simultaneously recorded physiological pressures. This synchronization pipeline enables the unified analysis of posture and physiological pressures along a common temporal axis. To demonstrate these dynamic tracking sequences in real time, three video recordings are provided as Supplementary material. To illustrate the interpretive value of synchronized posture and pressure data, Figure 5 presents three representative sequences spanning distinct postures and posture transitions. During sitting, posture remained constant and all pressure signals were correspondingly stable. During the walking-down sequence, transient fluctuations in abdominal pressure channels coincided with the descent portion of the sequence, suggesting a mechanical association that would not be identifiable from either signal alone. Finally, the kneeling sequence captures a kneeling-to-standing transition, during which vascular and abdominal pressures shift markedly and concurrently with the change in posture. This coordinated shift, precisely localized in time via the synchronized posture estimate, demonstrates the pipeline's capacity to disambiguate posture-driven pressure changes from other physiological sources of variation.

**Figure 5:**
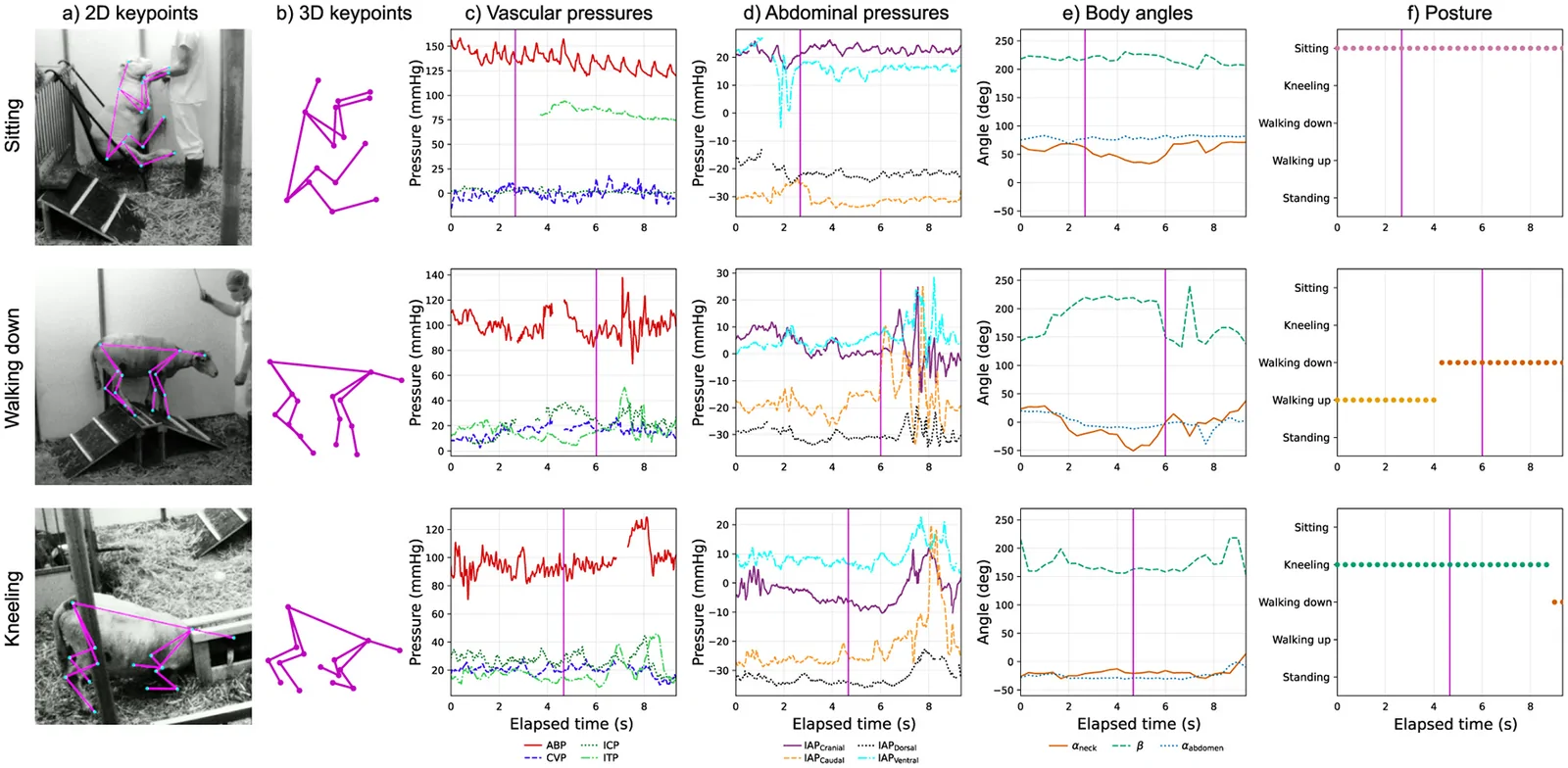
Overview of the outputs of the pipeline for three selected video sequences (sitting, walking up and down the brigde, and kneeling). In column **(a)** 2D keypoint estimation is shown over respective image, in column **(b)** the 3D skeleton obtained after trajectory smoothing, in column **(c)** vascular pressures plotted over time (ITP, intrathecal pressure; ICP, intracranial pressure; ABP, arterial blood pressure), in column **(d)** vascular pressures plotted over time (IAP_x_: intra-abdominal pressure for x = Cranial, Caudal, Dorsal, Ventral), in column **(e)** body angles plotted over time, and in column **(f)** body posture plotted over time. The magenta vertical dashed line indicates the time index of the frames depicted in columns **(a,b)**. Note the marked change in all pressure signals at the end of the kneeling sequence, the moment when the sheep stands up by first lowering the body, which is indicated as “walking down.” The video recordings for these sequences are provided as Supplementary material.

### 3.1 2D keypoints estimation

The results of the test set with 47 images on the KP detection were as follows: 77.4% for AP@[0.5:0.95], 100.0% for AP@0.5 and 85.1% for AP@0.75. The OKS scores for each KP are reported in Table 1, with the best scoring keypoint being the right carpal bones and the worst one being the head. The results of a comparison performed between Faster R-CNN + Lite-HR against SuperAnimal are shown in Table 2: SuperAnimal achieved an AP score of 7.0% for AP@[0.5:0.95] before fine-tuning. After fine-tuning for 400 training iterations on the same labeled frames as the ones used in the Faster R-CNN + Lite-HR approach, the 2D KP estimation reached a maximum AP of 76.4% for AP@[0.5:0.95], with the iteration that performs best selected for evaluation on the test set.

**Table 1:** Mean Object Keypoint Similarity (OKS) score for the fifteen anatomical keypoints and their kappa value (κ), evaluated on the test set (*n*=47).

Keypoint	Mean OKS	κ
head	0.808	0.025
neck	0.880	0.025
tail	0.881	0.025
phalanges	0.834	0.025
phalanges	0.905	0.025
phalanges	0.842	0.025
phalanges	0.853	0.025
carpal	0.872	0.025
carpal	0.932	0.025
tarsal	0.843	0.025
tarsal	0.861	0.025
elbow	0.850	0.025
elbow	0.895	0.025
patella	0.813	0.025
patella	0.844	0.025

**Table 2:** Comparison of mean Average Precision (AP), training budget, model size, and inference throughput for different models and training datasets.

Model	Super Animal (zero-shot)	Super Animal (fine tuned)	Faster R-CNN + Lite-HRNet
Training dataset	SuperAnimal	SuperAnimal + ours	Ours
AP@0.5 (%)	29.8	100.0	100.0
AP@0.75 (%)	0.0	93.6	85.1
AP@[0.5:0.95] (%) [95% CI]	7.0 [3.8, 10.4]	76.4 [72.1, 80.2]	77.4 [71.5, 82.8]
*n*	47	47	47
Parameters (M)	72.6	72.6	43.1
Inference (ms/frame, GPU)	243	243	146

AP computed via object keypoint similarity (OKS) with per-keypoint κ = 0.025, over *n* = 47 held-out test images (one instance per image); AP@[0.5:0.95] reported with 95% bootstrap confidence intervals (5,000 resamples over images). Inference timed on an NVIDIA RTX 3080 (batch size 1, native 1, 440 × 1, 080 resolution).

### 3.2 Triangulation

The accuracy of camera calibration through MASt3R was independently verified by manually annotating corresponding points across three representative frame pairs from the two camera views, yielding a mean reprojection RMSE of 1.77 pixels. Triangulation achieved a mean reprojection error of 16.39 px evaluated over our dataset (52 sequences, six sheep, 2,646 frame pairs), computed against the same 2D detections used as input. Triangulation additionally failed to produce a 3D estimate of at least one keypoint in 14.3% of frames, where a corresponding keypoint was undetected in at least one view.

### 3.3 Trajectory smoothing

An ablation study was performed to investigate the benefit of using the full trajectory estimation versus not including it in the pipeline, and the results are shown in Figure 6. The black skeletons represent detections obtained from frame-wise 3D KP estimation only, whereas the magenta skeletons show results of 3D KP estimation followed by trajectory smoothing. Incorporating temporal smoothing and skeletal constraints substantially improves the consistency of the reconstructed skeleton, as outlier KPs are reduced, limb lengths remain stable across frames, and missing joints due to occlusions are more reliably interpolated. Evaluated against the same detections used during optimization, trajectory smoothing yielded a mean reprojection error of 25.82 px, higher than frame-wise triangulation (16.39 px); this is expected, as triangulation directly minimizes per-frame reprojection error with no temporal or anatomical constraint, whereas trajectory smoothing trades a degree of per-frame reprojection fit for temporal continuity, anatomical plausibility, and robustness to missing detections. Bone-length dispersion, measured as the coefficient of variation of limb segment lengths across a sequence, was 28.1% under frame-wise triangulation versus 0% under trajectory smoothing; because the rigid-body formulation in trajectory smoothing constrains segment lengths by construction, this result demonstrates the instability of frame-wise triangulation rather than independently validating the accuracy of the trajectory smoothing reconstruction.

**Figure 6:**
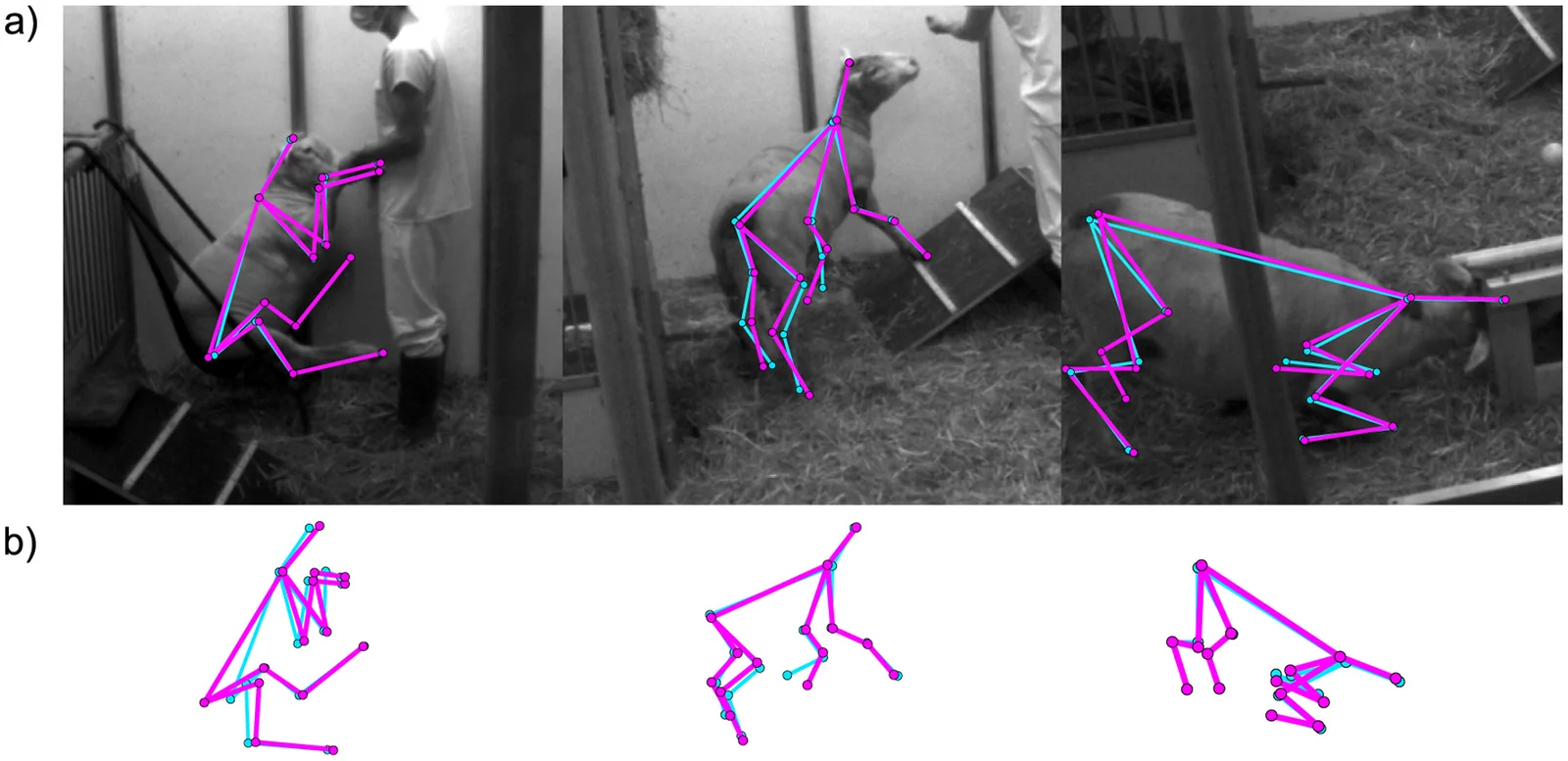
Ablation study between trajectory smoothing and frame-by-frame triangulation for three selected poses: sitting, standing and kneeling. **(a)** Camera frame with the reprojected skeletons overlaid: trajectory smoothing (magenta) vs. frame-by-frame triangulation (cyan). **(b)** Corresponding 3D skeleton reconstruction; the view is rotated 35° away from the camera view, to appreciate differences between the two 3D skeletons.

### 3.4 Pose classification

To evaluate the performance of the pose classifier, a confusion matrix (32) was created across the five posture classes and reported in Table 3. The randomized hyperparameter search identified a two-hidden-layer MLP with layer widths of 256 and 128 units (ReLU activations), L2 regularization strength α = 10^−3^, and a learning rate of 10^−3^ as the best configuration. The overall classification accuracy on the held-out test set reached 95.2%. Class-specific precision, recall, and F1 scores are summarized in Table 4. Performance was consistently high across all postures, with the lowest recall observed for Standing (90.9%), which was most frequently confused with Walking Down. Sitting was classified with near-perfect precision and recall (98.6% and 9.3%, respectively), consistent with its visually distinct configuration and larger representation in the training set (*n* = 142).

**Table 3:** Confusion matrix for the model on the held-out test set (*n* = 647 frames).

True\Pred.	Support	Standing	Walking up	Walking down	Kneeling	Sitting
Standing	209	90.9 (190)	1.0 (2)	6.2 (13)	1.0 (2)	1.0 (2)
Walking up	96	1.0 (1)	99.0 (95)	0.0 (0)	0.0 (0)	0.0 (0)
Walking down	114	4.4 (5)	0.0 (0)	95.6 (109)	0.0 (0)	0.0 (0)
Kneeling	86	4.7 (4)	0.0 (0)	0.0 (0)	95.3 (82)	0.0 (0)
Sitting	142	0.7 (1)	0.0 (0)	0.0 (0)	0.0 (0)	99.3 (141)

Each cell shows the row-normalized recall (%) with the raw frame count in parentheses; rows are true classes, columns are predicted classes.

**Table 4:** Precision, recall, and F1 score for each pose class, computed on the stratified held-out test set (*n* = 647 frames, 20% of the full dataset).

Metric	Standing	Walking up	Walking down	Kneeling	Sitting
Support (*n*)	209	96	114	86	142
Precision (%)	94.5 [90.1, 97.8]	97.9 [93.3, 100.0]	89.3 [78.5, 96.1]	97.6 [92.7, 100.0]	98.6 [94.1, 100.0]
Recall (%)	90.9 [85.4, 95.7]	99.0 [95.7, 100.0]	95.6 [87.9, 100.0]	95.3 [89.2, 100.0]	99.3 [96.8, 100.0]
F1-score (%)	92.7 [89.0, 95.7]	98.4 [95.4, 100.0]	92.4 [84.6, 96.8]	96.5 [91.9, 99.3]	98.9 [96.1, 100.0]

Bracketed values are 95% session-clustered bootstrap confidence intervals (44 recording sessions).

## 4 Discussion

In this study, we developed and validated a modular pipeline that enables automatic synchronization of multi-compartment physiological pressure recordings with markerless 3D posture estimates for a single subject in a chronic ovine model. To our knowledge, this is the first system to jointly address continuous posture quantification and telemetric pressure synchronization in this setting, providing a foundational tool for investigating posture-dependent pressure dynamics in large animals, while preserving natural, unconstrained posture through the use of markerless motion estimation, thereby supporting investigations on an animal model of conditions involving altered compartmental pressure dynamics. Our approach differs from prior sheep-specific pose work in scope rather than in individual components: methods such as (17, 18) classify posture directly from 2D detections, rather than estimating the 3D pose of the animal (Table 5).

**Table 5:** Leave-one-sheep-out cross-validation (6 folds; mean ± std across folds, pooled over five seeds), comparing logistic regression on the anatomical angles against a multi-layer perceptron (MLP) using world-frame vs. head-centered keypoint coordinates.

Classifier	Input	Accuracy (%)	Macro F1 (%)
Logistic regression	Anatomical angles	34.5 ± 8.6	31.8 ± 9.4
MLP	World-frame coordinates and anatomical angles	46.9 ± 13.1	32.4 ± 14.0
MLP	Head-centered coordinates and anatomical angles	68.8 ± 12.1	57.7 ± 22.3

### 4.1 Pose estimation performance

For 2D joint localization, we implemented a two-stage detector combining Faster R-CNN (21) for bounding box proposal with Lite-HRNet for KP estimation (22). We compared our approach with SuperAnimal (14), an off-the-shelf pre-trained model included in the DeepLabCut framework (12). Despite fine-tuning on our dataset, SuperAnimal underperformed relative to the Faster R-CNN + Lite-HRNet combination. This fact can be explained by the difference in input modalities: SuperAnimal is trained on standard three-channel color images, whereas our dataset consists of single-channel grayscale images. The results reported in Table 2 highlight the importance of species- and dataset-specific training in animal KP detection and caution against assuming that transfer learning will always outperform training from scratch under large domain shifts.

Our Faster R-CNN + Lite-HRNet 2D KP detector achieved a mean AP of 77.4%, and all KPs had an OKS score above 0.80. After evaluating the performance of the 2D detection model, it was subsequently re-trained on the entire dataset (train and test labeled images) to maximize the detection accuracy to be used in the pipeline. There are several factors that can influence the quality of KP predictions, including the quality and amount of training data and data augmentation, which is the process of systematically modifying labeled training images to increase the size of the dataset. As our custom training dataset contains augmented images from multiple sheep, minor differences between sheep size, shape, and position are not expected to affect the quality of KP predictions.

### 4.2 3D reconstruction and trajectory smoothing

Following 2D KP detection, the joint positions were triangulated into 3D coordinates. Because inaccuracies in 2D detection can lead to large errors in 3D coordinate estimation (33), and because anatomical plausibility requires limb lengths consistent with the sheep skeleton, we adapted the trajectory smoothing algorithm from AcinoSet (16) to a rigid skeleton with 15 joints. Although the original Acinoset3D method was validated on synthetic 3D data with controlled Gaussian noise, ground-truth 3D positions were not available in our dataset. Evaluation therefore relied on qualitative assessment of anatomical realism (joint lengths and angles) alongside the ablation study shown in Figure 6. Despite operating with fewer camera views (2 vs. 6) and a lower frame rate (3 FPS vs. 120 FPS) compared to the original method presented in Acinoset3D, our adapted trajectory smoothing produced visibly more realistic joint configurations than frame-wise triangulation alone.

Our results highlight the advantage of using temporal continuity to obtain anatomically plausible and complete posture estimates, even in the presence of partial occlusions or noisy detections. This demonstrates the suitability of trajectory smoothing for real-world scenarios where extensive camera setups or high-speed recording are not practical. Despite trajectory smoothing introducing additional computational overhead, the increased complexity remains compatible with offline analysis of pre-recorded data, making the method suitable for extended experimental sessions where real-time processing is not required.

### 4.3 Posture classification

The 3D KPs and derived anatomical angles were used to classify each frame into a predefined posture category, providing a compact overview of the full dataset. The primary benefit of using a pose classifier is that it provides a compact, queryable summary of the entire recorded dataset. The evaluation of the pipeline was limited on the poses included during training, which focused on those likely to affect physiological compartmental pressures and on postures that have clear human analogs, such as sitting or standing upright. Free behaviors such as lying or grazing were excluded from the current classifier, as they were outside the scope of this investigation. However, the classification framework is easily extensible: additional postures can be incorporated by labeling representative frames and retraining the MLP classifier. Posture classification into predefined categories allows the segmentation of video sequences, facilitating retrospective analysis, and simplifying future research.

### 4.4 Generalizability and design considerations

The true value of this pipeline lies in its generalizability to different subjects and trial conditions. Although the presented 3D joint and body-pose estimation results are based on data from a limited number of sheep, the pipeline is designed to operate on sheep not included in the training dataset without requiring additional labeling and retraining. The annotated 2D KPs and posture labels encompass frames from six sheep, providing sufficient appearance variability to support generalization given the low inter-individual variation within the species.

A key advantage of the proposed pipeline is its modular design, which supports flexible integration of future advances in KP estimation. If simultaneously acquired pressure and synchronized stereo image data are available, this pipeline can be used with minimal adjustments and is applicable to any long-term experiment in which posture modulates physiological pressures. Currently, the system records eight pressure signals that were selected because they were critical to investigations of cerebrospinal fluid dynamics. However, the pipeline is not intrinsically tied to this specific set of measurements. Pressure channels are treated generically throughout the acquisition, synchronization, and processing stages, allowing the number and type of recorded pressures to be easily adapted to other experimental requirements.

###  Limitations

Some limitations should be acknowledged. First, the pipeline is currently designed to operate on video sequences featuring only one animal visible in the stable during training. In practice, experimental sheep are housed with a companion animal,which can cause prolonged occlusions or multi-animal ambiguity. If this pipeline was to be applied continuously throughout the entire period that the sheep are in the stable to assess physiological pressures, this limitation becomes critical (11). In addition, future iterations will integrate top-down multi-animal identity tracking frameworks (19) combined with a bounding-box re-identification routine. This will allow the system to maintain individual sheep identities and seamlessly resume KP estimation even after prolonged total occlusions caused by the animal handler entering the frame.

Second, because continuous high-frame-rate video storage was not feasible, temporal interpolation was required to align 3D keypoints with the 100 Hz pressure recordings. This process generates artificial intermediate postures that may not fully reflect the true movements of the animal. During rapid transitions, assuming linear interpolation leads to an underestimation of joint velocity during the transition interval, and the precise onset of the associated pressure response cannot be resolved within the 333 ms inter-frame interval. The need for a low frame rate raised from video storage size constraints. However, our pipeline can be generalized to higher or lower sampling rates for the video system and for the pressure recordings, which would mitigate the transient uncertainty in future studies, provided they are conducted at a higher sampling frequency.

### 4.5 Future directions

Future work will focus on extending the system to support multiple animal detection, as well as introducing additional classes in the pose classifier. Future iterations will integrate top-down multi-animal identity tracking frameworks (19) combined with a bounding-box re-identification routine. This will allow the system to maintain individual sheep identities and seamlessly resume KP estimation even after prolonged total occlusions caused by the animal handler entering the frame. By adapting the 2D KP detection module, labeling new poses and retraining the MLP classifier, the pipeline can be extended to video sequences involving two or more freely moving sheep without trainer intervention, supporting physiological observations in natural behavioral settings and with low levels of stress for the animal. Together, these extensions will enable continuous, unsupervised posture monitoring throughout chronic ovine trials, supporting the investigation of naturally occurring posture transitions and their effects on cerebrospinal fluid dynamics and pressure regulation.

## 5 Conclusion

Our modular pipeline enables physiological pressure recordings to be automatically synchronized with the 3D joint positions and postures of an ovine model during training. Although ground-truth 3D data is not available for quantitative validation, qualitative observations indicate that, by leveraging trajectory smoothing and skeletal priors, this pipeline offers more consistent 3D keypoint reconstructions than just using triangulation of the 2D keypoints from the stereo camera views. The posture classification module enables efficient segmentation of large datasets into predefined posture categories, supports analysis, and facilitates further research into physiological dynamics. In general, the pipeline provides a flexible, open, and extensible framework for processing physiological and posture-related data in ovine studies. By enabling synchronized analysis of body posture and multi-compartment pressure dynamics under chronic conditions, this tool advances the understanding of large animal physiology and supports the translational investigation of posture-dependent pressure regulation relevant to research on cerebrospinal fluid dynamics.

## Data Availability

The datasets presented in this study can be found in online repositories. The names of the repository/repositories and accession number(s) can be found at: https://doi.org/10.3929/ethz-c-000790469.
